# Hospitals and the Law of Negligence

**Published:** 1910-05-14

**Authors:** 


### SPECIAL ARTICLES.

### HOSPITALS AND THE LAW OF NEGLIGENCE.

The liability of hospitals and hospital authorities in actions for negligence were matters which formed the subject of a paper at a recent meeting of the Medico-Legal Society in which Mr. Ross Brown, who opened the proceedings, called attention to all the leading cases on the subject. The position of the hospital appears to be altering somewhat. The general public regards access to a hospital as a matter of right. Among high and low the belief is prevalent that hospital authorities are under some obligation to minister to the sick and suffering. To raise the mild protest that hospitals are purely charitable institutions would appear, in these days, to be rather out of date. The fact, however, that a hospital is a charitable institution, and that the treatment is gratuitous, makes no difference when we come to consider the question of negligence. It is manifest that when a charge of negligence is preferred against a hospital, the governors can only be held liable if the damage is caused by some person for whose acts or omissions they are responsible; but the mere fact that the nurse or doctor whose carelessness has caused the injury is unpaid does not affect the question. Again, it does not matter whether the sufferer has been a consenting party to the operation or treatment. Suppose an injured person is suddenly brought from the street to the nearest hospital, he is not in a position to assent to the treatment of any particular person. Yet that person is liable if he be negligent. This is founded on the old case of Gladwell *v.* Steggall (1839), where it was held that a child was entitled to sue a clergyman who also practised as a doctor, although he had been employed by the child's father.

### What is Negligence?

What amounts to negligence in a doctor or nurse? We must solve that problem before the negligence of the hospital authorities need be considered. The law has been stated in the following terms: " Every person who enters a learned profession undertakes to bring to the exercise of it a reasonable degree of care and skill. He does not undertake, if he is an attorney, that at all events you shall gain your case; nor does a surgeon undertake that he will perform a cure; nor does he undertake to use the highest possible degree of skill. There may be persons who have higher education and greater advantages than he has; but he undertakes to bring a fair, reasonable, and competent degree of skill."

The liability of a surgeon does not, as we have seen, depend upon the question whether he is or is not to be remunerated; and the fact that he undertakes to perform an operation by himself or by his assistant does not constitute a guarantee of the success of such operation; but he is personally responsible for the act of his assistant and for the injury caused by the latter owing to want of skill (Hancke *v.* Hooper, 1835, 7 C. and P. 81). A medical man is not answerable merely because some other practitioner might possibly have shown greater skill and knowledge, or even used some greater degree of care, so long as he showed what, in the opinion of the jury, was due care and a competent degree of skill and knowledge (Rich *v.* Pierpont, 1862, 3 F. and F. 35).

It is hardly necessary to discuss the question of the liability of an unqualified practitioner, inasmuch as the quack is not allowed to show his nose inside the door of a hospital. As to criminal liability, we need only cite the following passage from Hales' " Pleas of the Crown ":—

" If a physician gives a potion to a person without any intent of doing him any bodily hurt, but with an intent to cure or prevent a disease, and, contrary to the expectation of the physician, it kills him, this is no homicide, and the like of a surgeon. And I hold the opinion to be erroneous that think, if he be no surgeon or physician, that occasioneth this mischance, that then it is felony, for physic and salves were before licensed physicians and surgeons; and therefore if they be not licensed, they are subject to the penalties in the statutes, but God forbid that any mischance of this kind should make any person not licensed guilty of murder or manslaughter."

Criminal liability may, however, be dismissed because it cannot extend beyond the persons themselves; it could never extend to the governors of a hospital.

### Institutional Liability.

Assume that the patient has got over his first stile, and has proved that a doctor or a nurse has been guilty of negligence, can the institution be held liable? It may be asked—Why should he attack the hospital? Why should he not content himself with an action for damages against the doctor or the nurse? The answer to this is that the litigant generally wants to fasten liability on the defendant who has most wool on his back. The liability of the hospital for the acts of doctor or nurse is a question of agency. The principal is liable, as a well-known text-book puts it, " for all the misfeasances and omissions of duty of his agent, in the course of his employment, although the principal did not authorise, or justify, or participate in, or indeed know of such misconduct, or even if he forbade the acts or disapproved of them. In all such cases the rule applies *respondeat superior*, and it is founded upon public policy and convenience, for in no other way could there be any safety to third persons in their dealings, either directly with the principal, or indirectly with him through the instrumentality of agents. In every such case the principal holds out his agent as competent and fit to be trusted, and thereby, in effect, he warrants his fidelity and good conduct in all matters within the scope of his agency."

Strange to say, there is no English case in which a hospital has been held liable for negligence. Indeed, in *Evans* v. *Liverpool Corporation* (1906), 1 K.B. 160, Mr. Justice Walton expressly decided that a rate-supported hospital could not be sued.

In this case a father sued the Corporation for damages because his son was discharged too soon from their infectious hospital, and conveyed scarlet fever to his other children. The following extract will show the grounds of the decision:—

"They do not undertake the duties of medical men or to give medical advice, but they do undertake that the patients in their hospitals shall have competent medical advice and assistance, and it is admitted that Dr. Archer was a competent medical man, and that no blame attaches to the defendants for employing him. Assuming that he made a mistake, even a negligent mistake, I do not think that the defendants are liable for its consequences; they have done all that the parent himself could have done, had he been able to have his son treated in his own house; he could not have done more than provide a proper home for the boy, and provide nurses and good medical attendance. The defendants have not failed in any of these respects, and I think that they are not liable for the mistake, if there was a mistake, of Dr. Archer."

We see no reason why this principle should not apply with equal force to a voluntary hospital; and there is a still more recent case which shows that there is at least a duty imposed upon the authorities of a hospital to use due care in selecting their staff. We refer to *Hillyer* v. *St. Bartholomew's Hospital* (25 T.L.R. 762). It was there laid down that the only duty undertaken by the governors of a public hospital towards a patient who is treated in the hospital is to use due care and skill in selecting their medical and nursing staff. The physicians and surgeons who give their services at the hospital are employed to exercise their profession to the best of their abilities according to their own discretion, but in exercising it they are in no way under the orders, or bound to obey the directions, of the governors. Although the nurses employed at the hospital are servants of the governors for general purposes, they are not so for the purposes of operations conducted by the medical staff."

The plaintiff sued the defendants to recover damages for injuries which he alleged he had sustained through the negligent conduct of an operation at the hospital. The operation was conducted by a consulting surgeon, who was attached to the hospital but was not under the control of the defendants. During the operation he was assisted by members of the surgical and medical staff. It was held that the action was not maintained against the defendants. The facts, therefore, were against the plaintiff; but if there had been negligence proved the hospital might have been held liable. "It is now settled," said Lord Justice Farwell, "that a public body is liable for the negligence of its servants in the same way as private individuals would be liable under similar circumstances, notwithstanding that it is acting in the performance of public duties like a local board of health, or of eleemosynary and charitable functions like a public hospital."

The fact that this article is barren of much direct authority upon the subject under discussion affords a striking testimony to the efficiency of our great hospitals.

One point which affects rate-aided hospitals—*e.g.* workhouse infirmaries—may be noted in conclusion. Any action for negligence against such an institution would have to be brought within six months, and if the plaintiff proved to be unsuccessful he might be ordered to pay the costs of the defendants as between solicitor and client. This is a meed of protection which is not afforded to those who are in control of ordinary hospitals.

